# The correlation between resilience and mental health of adolescents and young adults: a systematic review and meta-analysis

**DOI:** 10.3389/fpsyt.2025.1536553

**Published:** 2025-02-10

**Authors:** Shulai Luo, Jiangtao Hu, Junshuai Zhang, Zhengyang Mei, Zhongjian Tang, Shi Luo

**Affiliations:** School of Physical Education, Southwest University, Chongqing, China

**Keywords:** resilience, mental health, adolescents, young adults, meta-analysis, correlation

## Abstract

**Background:**

Adolescents and young adults (AYAs) aged 10–25 exhibit an increased prevalence of mental health disorders. Resilience has been well established as a positive factor in promoting and protecting mental health. This systematic review and meta-analysis aimed to quantify the correlation between resilience and mental health in AYAs by including relevant observational studies. Additionally, it explored potential moderators such as percentage of female participants, sample regions, and resilience measurements.

**Methods:**

A comprehensive search of the PubMed, Embase, Cochrane Library, Web of Science and Scopus databases was conducted until September 2024. CMA 3.0 software was used to perform meta-analysis, publication bias and sensitivity analysis of the included studies, and the moderating effect was verified by meta-analysis of variance (ANOVA).

**Result:**

Nineteen studies involving a total of 17,746 participants were included, and the summary effect sizes from the random effect model showed that resilience among AYAs had a correlation coefficient of -0.391 with negative indicators of mental health (95% CI: - 0.469, - 0.308, p < 0.001), and a correlation coefficient of 0.499 with positive indicators of mental health (95% CI: 0.400, 0.586, p < 0.001). Additionally, sample regions and resilience measurements significantly moderated the correlation between resilience and positive indicators of mental health.

**Conclusion:**

Resilience in AYAs showed a moderately negative correlation with negative indicators of mental health and a moderately strong positive correlation with positive indicators of mental health. The findings strengthened the basis for future resilience research in AYAs aged 10–25, highlighting the potential of resilience to help mitigate the increasing mental health challenges faced by this population.

## 1 Introduction

Recent statistics from the World Health Organization showed that the prevalence of diagnosed mental health disorders among adolescents and young adults (AYAs) aged 10-25 ranged between 10% - 20% (1). Mental health disorders refer to health conditions that are characterized by significant changes or disturbances in emotion, thinking, or behavior (2). The 10-25 age group encompasses adolescents aged 10-19 and young adults aged 18-25 (3, 4), where mental health disorders drawn increasing public attention and are now recognized as a critical global public health issue (5, 6). In specific, approximately one in seven adolescents aged 10-19 suffers from mental health disorders, mainly anxiety and depression, which account for 13% of the global burden of disease in this age group (1). Among young adults aged 18-25, the prevalence of major depressive episodes has risen sharply, increasing from 8.8% in 2005 to 15.2% in 2019, and the percentage of people with severe impairment from major depressive episodes nearly doubling from 5.2% to 10.3% during the decade from 2009 to 2019 (7). Without timely intervention, these mental health disorders can result in long-lasting adverse effects across individuals including social isolation (8), unemployment (9), and substance abuse (10), while significantly increasing the incidence of suicidal behaviors among AYAs (11). Alarmingly, suicide has now become the second leading cause of death among individuals aged 15-25 (12). The consistent epidemiological evidence indicates that all major syndromes constituting approximately 75% of mental health disorders begin before the age of 25 (13). Therefore, prevention and intervention mechanisms for mental health disorders in AYAs aged 10-25 are urgently needed to effectively reduce the disease burden.

Building resilience has received considerable attention from researchers due to its crucial role in reducing the risk of mental health disorders and promoting individual mental health (14). The conceptualization and study of resilience initially emerged from research on children at high risk for severe psychopathology (15). Over time, resilience has been defined in various ways, including as a personality trait that enables an individual to cope with adversity and to achieve positive adjustment and development (16, 17), or as a functional or behavioral outcome that overcomes and helps an individual to recover from adversity (18, 19). However, we propose a broader used definition of resilience as the ability and dynamic process of maintaining or regaining mental health despite experiencing adversity (20–22). Notably, many empirical studies have demonstrated its effectiveness in coping with stress and facilitating positive adaptations to protect the mental health of individuals (23–28). Meanwhile, researchers have explored the theoretical mechanisms underlying this positive effect, developing models of the relationship between resilience and mental health that incorporate mediating variables such as positive affect, perceived social support (29), and coping strategies (30). Despite substantial theoretical and empirical evidence supporting the relationship between resilience and mental health (31–33), previous reviews have paid relatively limited attention to AYAs, leaving the precise strength of this association unclear. In fact, individuals aged 10-25 experience an increased biological stress response (34) which makes them a highly susceptible group to various stressors (35). As a result, this age group exhibits a dramatically increased prevalence of mental health disorders (36). Examining the correlation between resilience and mental health specifically within AYAs will be beneficial in informing targeted resilience interventions. In addition, the dual-factor model of mental health (37, 38) emphasizes the need to evaluate mental health comprehensively. This entails considering both the absence of negative psychopathological indicators and the presence of positive psychological indicators such as subjective well-being, life satisfaction (39, 40). However, previous reviews have rarely explored these aspects in an integrated manner. To address this gap, our study attempts to conduct a more comprehensive quantitative review that combines these dimensions, with a particular focus on AYAs.

Potential moderators influencing the relationship between resilience and mental health in AYAs warrant further investigation. Building on previous studies, we focus on examining the moderating effects of gender, sample regions, and resilience measurements. First, regarding gender, researchers have found that as the percentage of female participants increases, the association between resilience and positive indicators of mental health becomes stronger (41). Second, results observed in samples from different regions often vary due to multiple influencing factors, such as sociocultural differences between Western and Eastern societies or disparities in the stages of resilience research (42, 43). These factors may contribute to differences in the strength of the relationship between resilience and mental health indicators. Lastly, the diversity of resilience measurements, with their different measurement properties, may influence the association between resilience and mental health indicators, resulting in variations in the observed outcomes (44).

In summary, this study aims to systematically quantify the correlation between resilience and both positive and negative indicators of mental health in AYAs through a review of the literature, along with an exploration of some potential moderators including percentage of female participants, sample regions, and resilience measurements.

## 2 Methods

This systematic review was conducted following the Preferred Reporting Items for Systematic Reviews and Meta-Analyses (PRISMA) guidelines, and was pre-registered in the International Prospective Register of Systematic Reviews (PROSPERO; ID: CRD42024604631).

### 2.1 Search strategy

We performed a systematic search in the following five electronic databases (PubMed, Embase, Cochrane Library, Web of Science, and Scopus) and used a snowball strategy to find relevant articles from their references and subsequent citations. The search of literature was limited to the period covered from the inception of each database till September 2024. The detailed search strategy is provided in the **Supplementary Material**, as the PubMed interface.

### 2.2 Inclusion and exclusion criteria

The retrieved studies were included in the meta-analysis when the following criteria were met: (1) published studies in English; (2) the study types were observational studies, which included cohort studies, case-control studies, cross-sectional studies and longitudinal studies reporting multiple cross-sections; (3) the participants were AYAs aged 10-25 years; (4) measured resilience, negative indicators (including symptoms of psychopathology and negative affect such as anxiety, depression, schizophrenia, bipolar and other reported mental health disorders or problems) or positive indicators (including subjective well-being, life satisfaction, quality of life etc.) of mental health; (5) reported Pearson’s correlation coefficients (r) between resilience and the above indicators of mental health.

The exclusion criteria are as follows: (1) newspaper, conference presentations, and review literature; (2) studies with incomplete or unreported data; (3) non-observational studies and purely descriptive studies.

### 2.3 Literature screening

Endnote X9 literature management software was used to detect and exclude all the duplications. Then, two authors evaluated the titles and abstracts of the remaining articles to ensure their eligibility for inclusion in the study. No further review was conducted for articles that met the exclusion criteria. After that, the two authors reviewed the full texts of the eligible literature. During this process, any disagreements were discussed to reach a resolution, or addressed by consulting another author.

### 2.4 Data extraction and quality assessment

After literature screening, the two authors reviewed the full text for data extraction. The following information of each included study was extracted: author, country, mean age, gender, resilience measurements, and mental health measurements of both negative and positive indicators, as well as Pearson’s correlation coefficient between resilience and both indicators of mental health. Separate extractions were performed if several different samples were investigated in the same study.

The Joanna Briggs Institute (JBI) Critical Appraisal Checklist for Analytical Cross-Sectional Studies was applied for quality assessment (45). This checklist consists of 8 items, each of which includes four answers (“yes,” “no,” “unclear,” and “not applicable”). Each “yes” answer corresponds to one point and the rest of the answers are assigned no points, with the points summed to give a total score for each study. Converting the total score to percentages, we rated studies scoring 70% and above as high quality, studies scoring 50% to < 70% as moderate quality, and studies scoring less than 50% as low quality (46).

### 2.5 Data analysis

All statistical analyses of our study were performed using Comprehensive Meta-Analysis version 3.0 (CMA 3.0) software. Pearson’s correlation coefficient was utilized to evaluate the correlation between resilience and both negative and positive indicators of mental health. First, we converted r values to Fisher’s Z by using the formula Fisher’s Z = 0.5 ln [(1 + r)/(1-r)]. The values obtained were then weighted according to the sample size using the formula SEz = (1/(n-3))^1/2^. Finally, all values were converted to r by the formula Summary r = (e^2z^ – 1)/(e^2z^ + 1) to evaluate the correlation between resilience and mental health. In addition, according to (47), r = 0.1 represented low correlation, r = 0.3 represented moderate correlation, and r = 0.5 and above represented strong correlation. All mean effect sizes were calculated by averaging the correlation coefficients of all independent samples, weighted by their inverse variance. Heterogeneity test was conducted using Cochran’s Q and I^2^ statistics (48). The fixed effect model was used to summarize the effects when I^2^ < 50%; otherwise, the random effect model was more appropriate for the analysis of the effect sizes from the existing literature (49). Moreover, a meta-analysis of variance (ANOVA) was used on categorical variables to test whether it was possible to moderate the correlation between resilience and mental health. The moderating variables in this study were identified based on insights from the existing literature, including the percentage of female participants, sample regions, and resilience measurements. The differences between and within groups were assessed by the Q test, and groups with fewer than 3 studies (k < 3) were removed due to concerns regarding under-representation and limited statistical reliability. Funnel plots and Egger’s linear regression (50) were used to evaluate publication bias. Sensitivity analysis was also conducted to test the robustness of the results of this study.

## 3 Results

### 3.1 Literature selection

**Figure 1:**
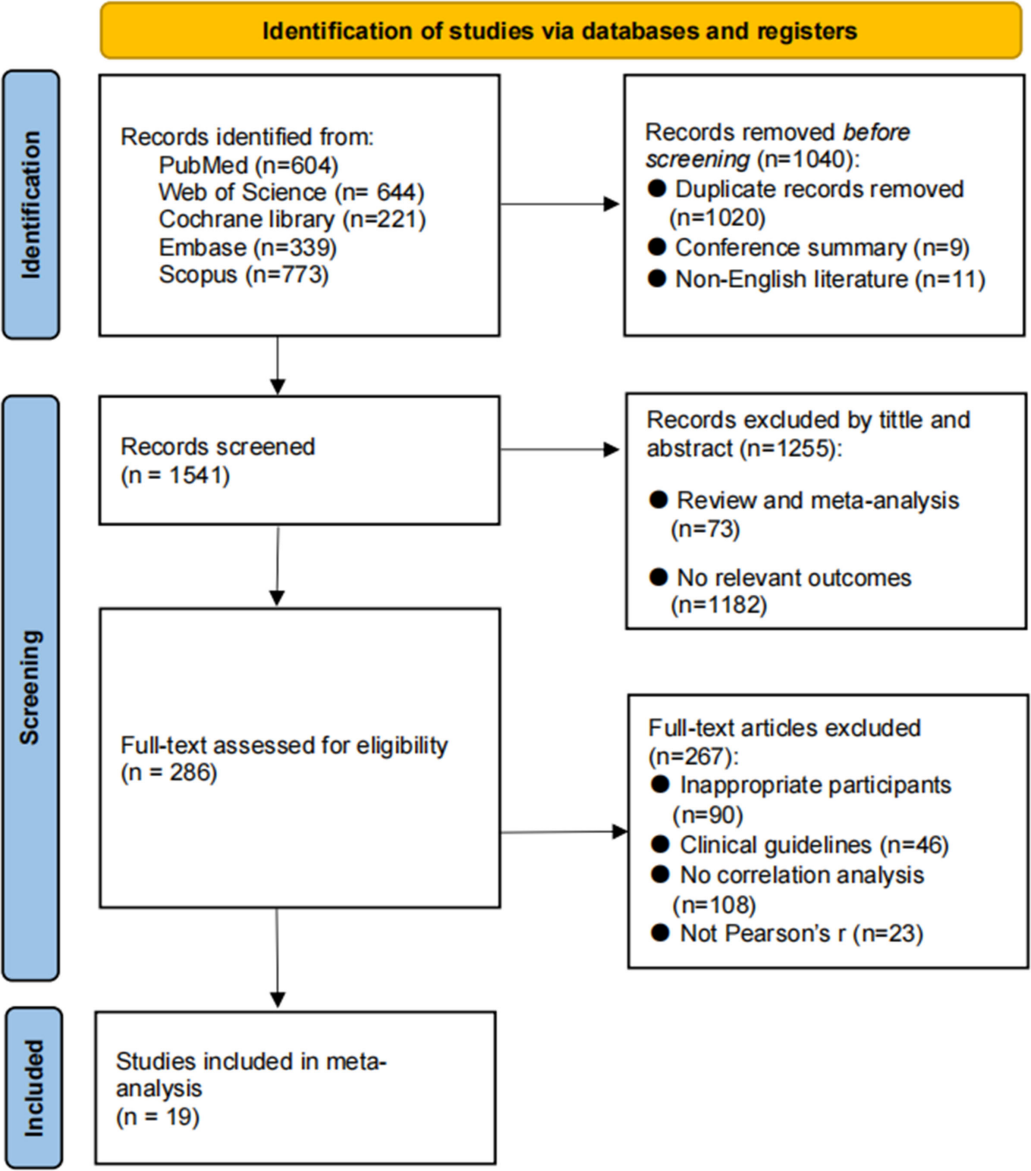
The process of literature screening.

We retrieved 2581 records from the database in the initial literature search. After removing 1020 duplicates, 11 non-English literature and 9 conference abstracts, 1541 studies were retained. Title and abstract screening excluded 1255 articles that did not match our requirements in terms of sample or topic. The remaining 286 articles were screened in full text, and 267 articles were excluded due to unavailability of data, inappropriate article type, and not reporting Pearson’s correlation coefficient. Finally, a total of 19 studies were included in the current review. **Figure 1** shows the flow chart of literature selection process.

### 3.2 Study characteristics

The 19 included studies, involving a total of 17746 participants, were conducted between 2011-2024, with more than half of them conducted in the last three years. The detailed characteristics of the included studies are shown in **Table 1**. Five studies were from China (mainland and HK), three from Spain, two from Norway and Poland, and one each from Malaysia, Nigeria, Canada, Japan, Saudi Arabia, USA, Germany and India. All studies measured both resilience and mental health, and reported Pearson’s correlation coefficient. The CD-RISC was utilized in three studies, while four studies applied the short version (CD-RISC-10). Of the remaining studies, three utilized READ and RS, two used BRS, and PIES, PSS/GHQ, CYRM, CPYDS, RSCA. Out of the 19 included studies, twelve studies measured the negative indicators of mental health, including anxiety, depression, perceived stress, burnout, etc. However, thirteen involved the assessment of various positive indicators of mental health, such as quality of life, life satisfaction, psychological well-being, hope, optimism, self-efficacy.

**Table 1:** Basic characteristics of the included studies.

Study	Country (sample regions classification)	Mean age (range)	Total/male/female	Resilience measurements	Mental health indicators	Pearson’s r
Achour and Nor (51)	Malaysia (Eastern)	(15-19)	200/NA/NA	Psychosocial Inventory of Ego Strengths (PIES)	Life satisfaction (SLS)	0.431
Anyan et al. (52)	Norway (Western)	(13-17)	529/244/285	Resilience Scale for Adolescents (READ)	Depressive symptoms (SMFQ) Anxiety symptoms (53)	-0.204 -0.325
Chow et al. (54)	China (Eastern)	NA	678/170/508	Connor-Davidson resilience scale (CD-RISC-10)	Psychological well-being (WHO-5)	0.378
de la Fuente et al. (55)	Spain (Western)	21.33 (19-25)	1069/155/914	Connor-Davidson resilience scale (CD-RISC)	Behavioral positivity (EDP) Burnout (MBI)	0.592 -0.372
Hjemdal et al. (56)	Norway (Western)	16.4(14-18)	307/167/140	Resilience Scale for Adolescents (READ)	Anxiety (DASS-21) Depression (DASS-21) Stress (DASS-21)	-0.34 -0.39 -0.29
Ibigbami et al. (57)	Nigeria (Africa)	17.11(13-19)	1321/NA/NA	Connor-Davidson resilience scale (CD-RISC-10)	Depressive symptoms (PHQ-9)	-0.31
Las-Hayas et al. (58)	Spain (Western)	12.4	3727/1820/1907	Resilience Scale for Adolescents (READ)	Mental well-being (WEMWBS) Health-related quality of life (KIDSCREEN-10) Stress (PSS-4) Depression (PHQ-9) Generalized anxiety disorder (GAD-7)	0.710 0.731 -0.609 -0.596 -0.501
Lau (59)	China (Eastern)	21.56	125/63/62	Perceived Stress Scale (PSS), General Health Questionnaire (GHQ)	Anxiety (DASS‐anxiety, 7 items) Depression (DASS‐depression, 7 items)	-0.462 -0.58
Marulanda and Addington (60)	Canada (Western)	18.09	80/43/37	Child and Youth Resilience Measure (CYRM)	Depression (CDSS) Anxiety (SIAS and SAS)	-0.46 -0.34 -0.32
Masuyama et al. (61)	Japan (Eastern)	14.03 (12-15)	965/NA/NA	Bidimensional resilience scale (BRS)	Depressive symptoms (DSRS-C)	-0.51
Rayani et al. (62)	Saudi Arabia (Middle East)	NA	175/72/102	Connor-Davidson resilience scale (CD-RISC-10)	Perceived well-being (WHO-5)	0.281
Rew et al. (63)	USA (Western)	21.25 (18-24)	111/60/51	Resilience scale (RS)	Life satisfaction (SLS) Social Connectedness Optimism Hope	0.29 0.24 0.48 0.48
Scheiner et al. (64)	Germany (Western)	12.31 (11-14)	2154/1099/1055	Connor-Davidson resilience scale (CD-RISC-10)	Health-related quality of life (KIDSCREEN-10) Self-efficacy (RESE-R) Self-esteem (SISE) Depressive symptoms (PHQ-9)	0.61 0.52 0.58 -0.51
Shek and Liang (65)	China (Eastern)	12.59	3291/1719/1572	Chinese Positive Youth Development Scale (CPYDS)	Life satisfaction (SLS)	0.45
Shi et al. (66)	China (Eastern)	20.42 (18-25)	521/180/341	Connor-Davidson resilience scale (CD-RISC)	Life satisfaction (SLS)	0.47
Sia and Aneesh (67)	India (Eastern)	15.03 (13-17)	385/179/206	Brief Resilience Scale (BRS)	Psychological Well-being (PWBS)	0.217
Visier-Alfonso et al. (68)	Spain (Western)	20.27	370/62/308	Connor-Davidson resilience scale (CD-RISC)	Stress (SINS) Depressive symptoms (PROMIS) anxiety symptoms (PROMIS) Psychological well-being (SPWB)	-0.145 -0.32 -0.24 0.4
Zhu et al. (69)	China (Eastern)	(10-19)	1284/620/664	Resilience Scale for Chinese Adolescents (RSCA)	Mental health problems (MSSMHS)	-0.28
Konaszewski et al. (70)	Poland (Western)	Study 1: 15.71 (13-18) Study 2: 16.34 (13-18)	Study 1: 201/121/80 Study 2: 253/172/81	Resilience scale (RS-14)	Life satisfaction (SLS) Depression (KADS) Mental Well-being (WEMWBS)	0.65 -0.31 0.71

### 3.3 Effect size and heterogeneity

#### 3.3.1 The summary correlation between resilience and negative indicators of mental health

**Figure 2:**
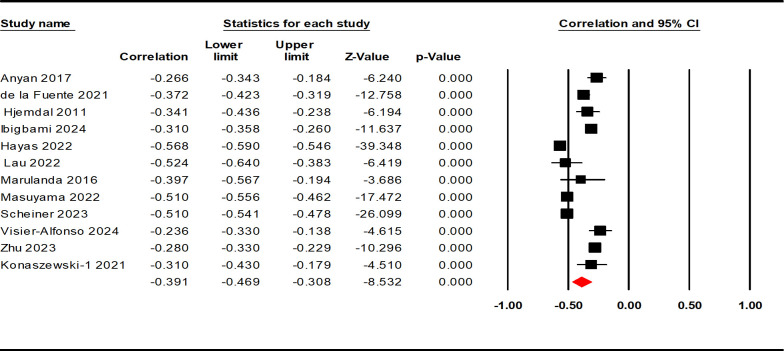
Forest plot of the correlation between resilience and negative indicators of mental health.

Data from 12132 AYAs were included in the 12 studies. The results of the heterogeneity test showed a high level of heterogeneity among the included studies (Q = 266.615, p < 0.001, I^2^ = 95.874%). Therefore, we calculated the mean weighted effect size (r), sample size (k), and 95% confidence intervals using a random effect model (**Table 2**). The results showed a moderately negative correlation between resilience and negative indicators of mental health (r = - 0.391, 95% CI: - 0.469, - 0.308, p < 0.001; see **Figure 2**).

**Table 2:** Random-model of the correlation between resilience and mental health.

Mental health	k	N	Mean r effect size	95% CI for r		Test of null (2-tail)		Homogeneity test				Tau-squared			
				LL	UL	*z*- Value	*p*-Value	Q_(T)_	df	*p*	*I*-squared	Tau-squared	SE	Variance	Tau
Negative indicators	12	12132	-0.391	-0.469	-0.308	-8.532	0.000	266.615	11	0.000	95.874	0.025	0.015	0.000	0.159
Positive indicators	13	13172	0.499	0.400	0.586	8.661	0.000	536.259	12	0.000	97.762	0.049	0.031	0.001	0.222

k, number of effect sizes; N, number of samples.

#### 3.3.2 The summary correlation between resilience and positive indicators of mental health

**Figure 3:**
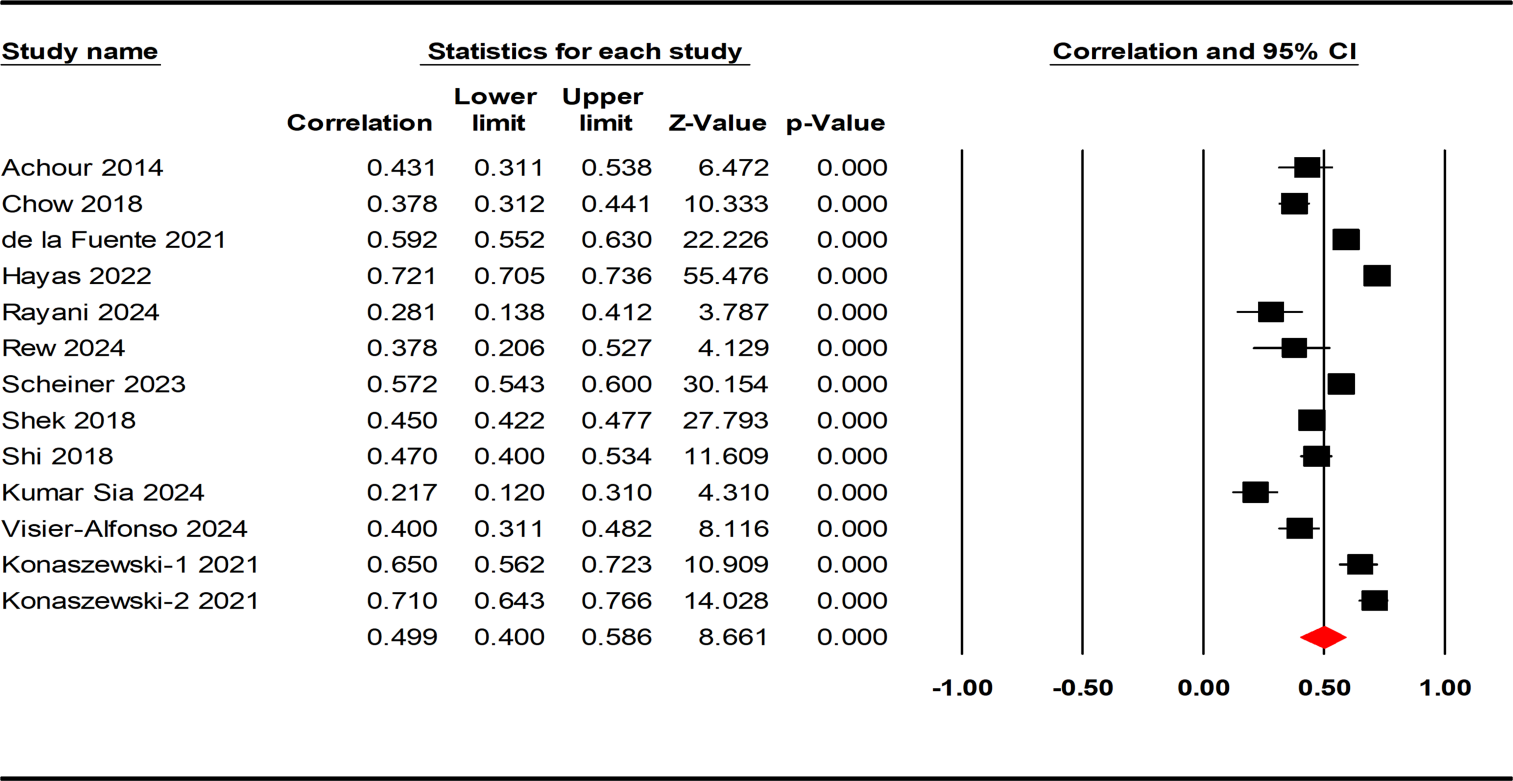
Forest plot of the correlation between resilience and positive indicators of mental health.

The correlations between resilience and positive indicators of mental health were reported in 13 studies, involving 13135 AYAs. The results of the heterogeneity test were similar to the negative indicators, demonstrating high heterogeneity (Q = 536.259; p < 0.001; I^2^ = 97.762%). The effect size calculated by the random effect model (**Table 2**) revealed a moderately strong positive correlation between resilience and positive indicators of mental health among the AYAs (r = 0.499, 95% CI: 0.400, 0.586, p < 0.001; see **Figure 3**).

### 3.4 Quality assessment

The quality of included cross-sectional studies was assessed using the JBI Critical Appraisal Checklist for Analytical Cross-Sectional Studies, which has been well-validated and considered the most commonly used tool for assessing bias in analytical cross-sectional studies (71). Two authors independently assessed the quality of each study, and any disagreements were resolved through discussions at group meetings. A total of 16 studies received scores above 70%, classifying the majority of the included studies as high quality. Among the remaining three studies, two were categorized as moderate quality, while one was classified as low quality. The detailed results of each included study are shown in the **Supplementary Material**.

### 3.5 Moderator analyses

The current study used meta-analysis of variance (ANOVA) to test the moderating effects of three variables below: percentage of female participants, sample regions, and resilience measurements.

Results from the moderator analyses are presented in **Tables 3**. In terms of the positive indicators of mental health, the sample regions and the resilience measurements significantly moderated the correlation between resilience and mental health (sample regions: Q_BET_ = 11.338, p < 0.001, resilience measurements: Q_BET_ = 69.932, p < 0.001). More specifically, Studies from Western countries reported the stronger correlation between resilience and mental health (Western: r = 0.589, 95% CI: 0.494, 0.670, p < 0.001, Eastern: r = 0.380, 95% CI: 0.304, 0.452, p < 0.001). For resilience measurements, the RS scale reported stronger correlations than CD-RISC scale and other scales (RS: r = 0.579, 95% CI: 0.335, 0.750, p < 0.001, CD-RISC: r = 0.467, 95% CI: 0.366, 0.557, p < 0.001, Others: r = 0.371, 95% CI: 0.212, 0.512, p < 0.001), while the READ scale group was excluded due to a small sample size of less than 3 studies (k=1). However, percentage of female participants did not have a significant moderating effect on the correlation between resilience and mental health. Regarding the negative indicators of mental health, none of the sample regions, percentage of female participants, or resilience measurements were found to significantly modulate the correlation (p>0.05).

**Table 3:** Moderators of the correlation between resilience and mental health.

Moderators	Between-group effect (Q_bet)_	k	N	Mean r effect size	95% CI for r		Homogeneity test within each group (Q_w_)
					LL	UL	
Percentage of female participants							
Negative indicators	0.529						
>50%		5	6959	-0.353^***^	-0.504	-0.182	199.913^***^
<50%		5	2867	-0.423^***^	-0.519	-0.316	21.282^***^
Positive indicators	2.807						
>50%		7	6925	0.457^***^	0.270	0.610	387.034^***^
<50%		5	6010	0.588^***^	0.466	0.689	93.587^***^
Sample regions							
Negative indicators	3.10						
Western		8	8437	-0.385^***^	-0.479	-0.282	156.613^***^
Eastern		3	2374	-0.439^***^	-0.597	-0.248	45.295^***^
Positive indicators	11.338^***^						
Western		7	7885	0.589^***^	0.494	0.670	166.165^***^
Eastern		6	5287	0.380^***^	0.304	0.452	32.331^***^
Resilience measurements							
Negative indicators	1.531						
CD-RISC (including short version)		4	4914	-0.365^***^	-0.480	-0.237	68.265^***^
READ		3	4563	-0.403^***^	-0.602	-0.155	80.843^***^
Others		4	2454	-0.430^***^	-0.568	-0.268	45.297^***^
Positive indicators	69.932^***^						
CD-RISC (including short version)		6	4967	0.467^***^	0.366	0.557	82.432^***^
RS		3	565	0.579^***^	0.335	0.750	26.681^***^
Others		3	3913	0.371^***^	0.212	0.512	23.918^***^

k, number of effect sizes; N, number of samples; ***p <.001.

### 3.6 Publication bias and sensitivity analysis

**Figure 4:**
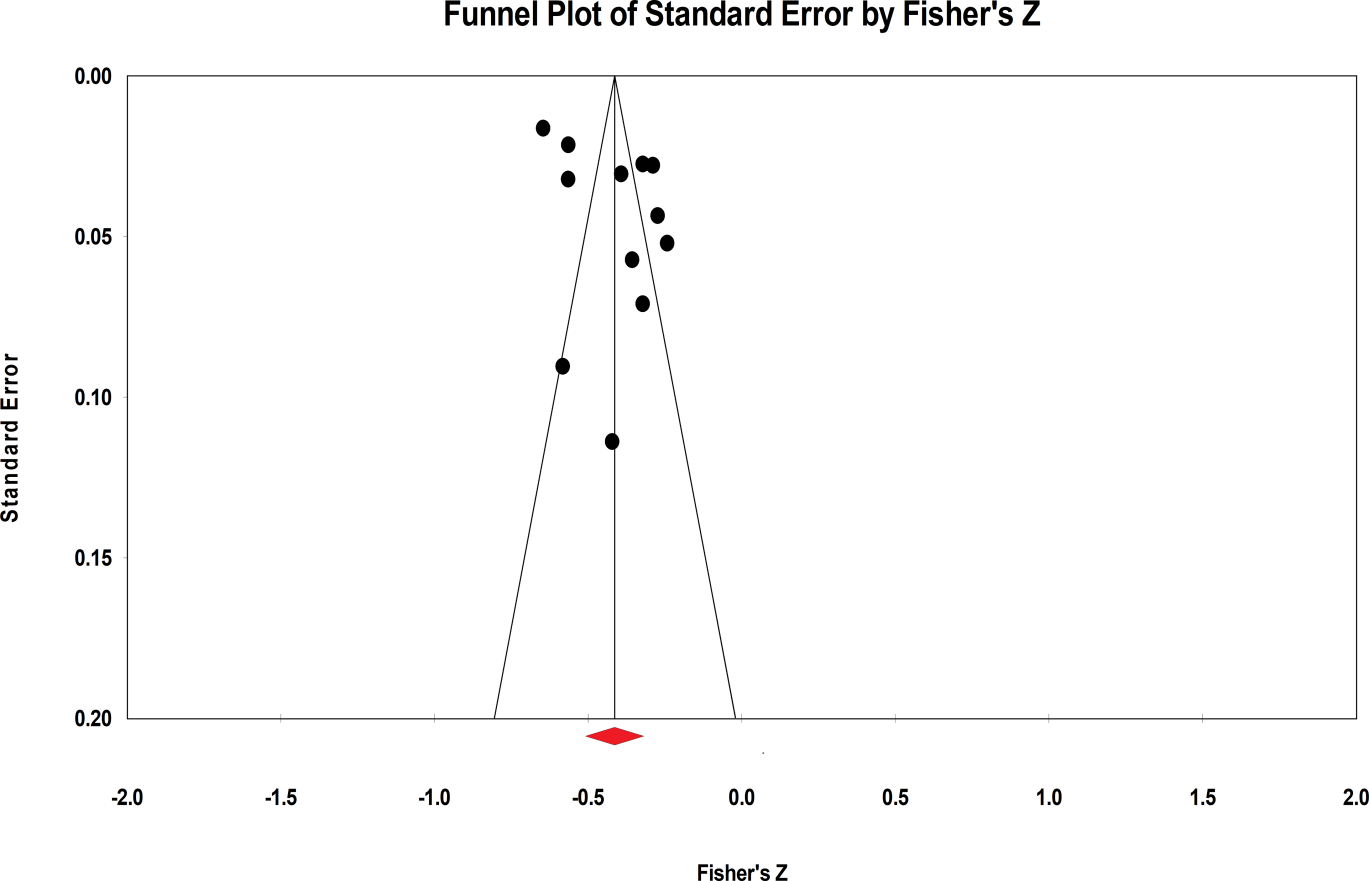
Funnel plot of the correlation between resilience and negative indicators of mental health.

**Figure 5:**
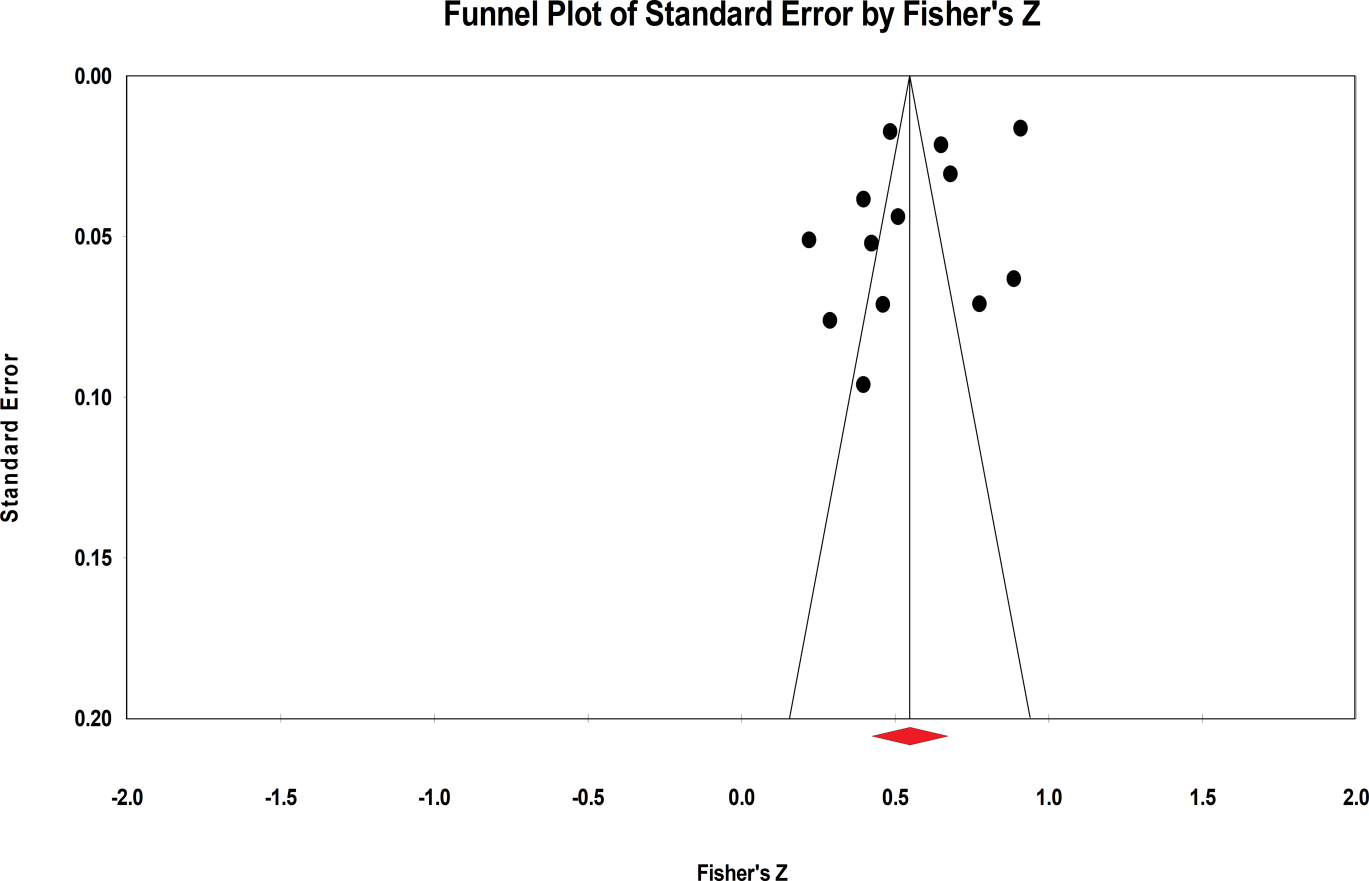
Funnel plot of the correlation between resilience and positive indicators of mental health.

**Figure 6:**
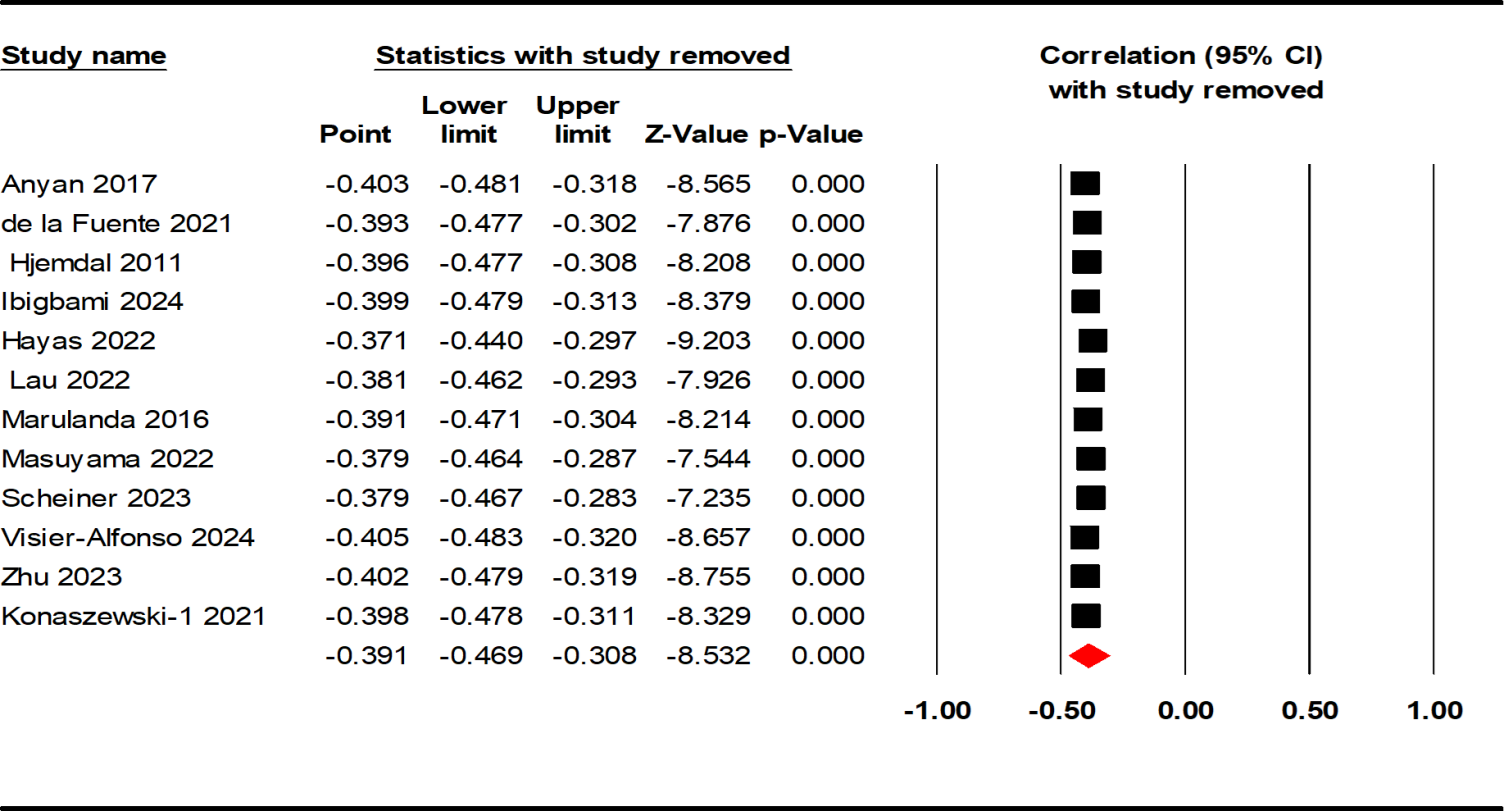
Sensitivity analysis of the correlation between resilience and negative indicators of mental health.

**Figure 7:**
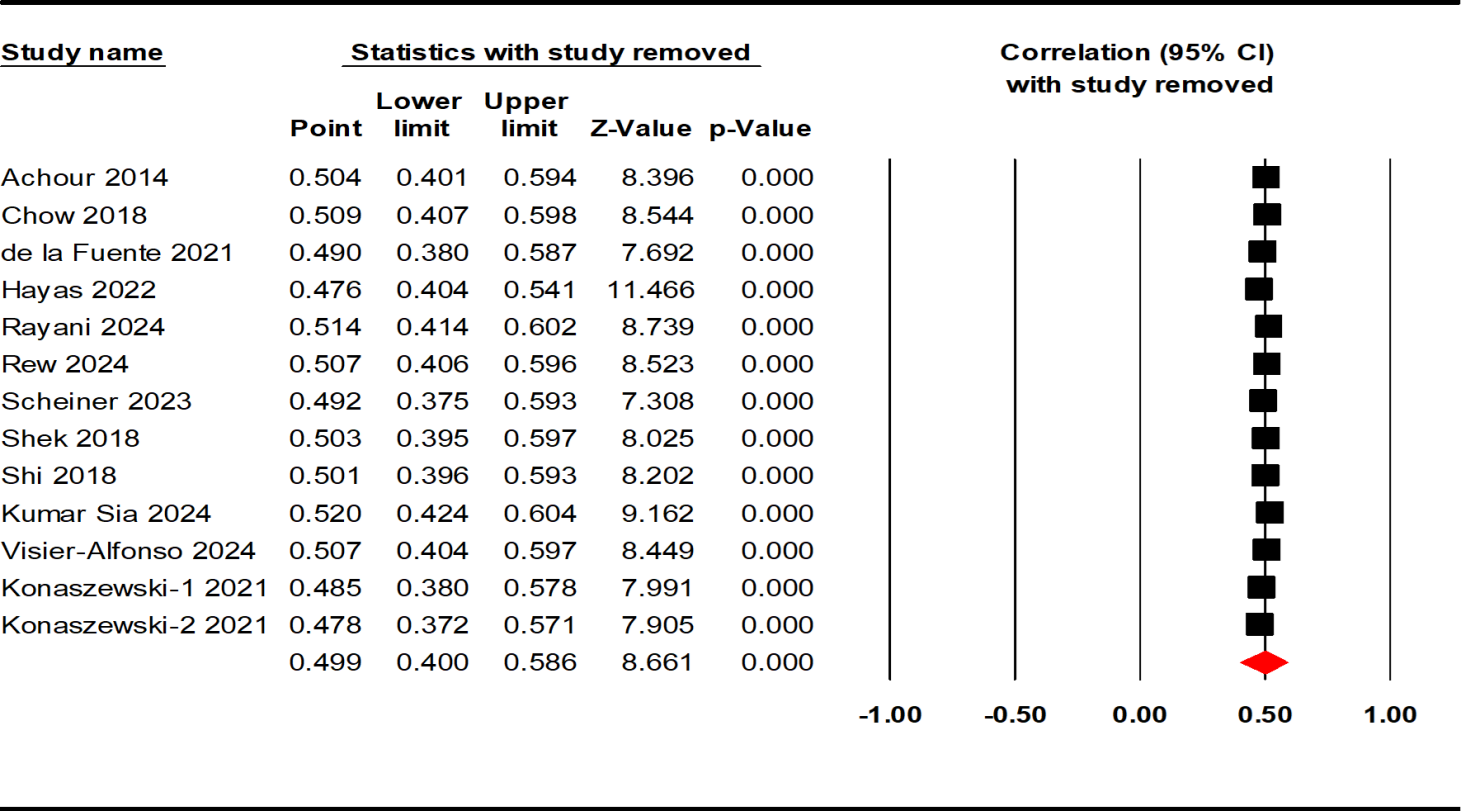
Sensitivity analysis of the correlation between resilience and positive indicators of mental health.

The present meta-analysis evaluated publication bias using funnel plots and Egger linear regression. The funnel plot provided insufficient evidence of the symmetrical distribution of effect sizes for both negative and positive indicators (**Figures 4**, **5**). Therefore, Egger linear regression was utilized to provide more reliable evidence. The p-values of the results indicate that there were no significant publication biases for both indicators (negative indicators: t = 1.965, p = 0.077; positive indicators: t = 1.545, p = 0.151). The sensitivity analysis was conducted by removing the included samples one by one, and a significant shift in the effect sizes would represent a lack of non-robustness. However, our results indicated that the effect sizes of resilience and both indicators of mental health in AYAs were stable, therefore, the results of this meta-analysis were robust (**Figures 6**, **7**).

## 4 Discussion

The current study aimed to systematically summarize the evidence on the correlation between resilience and both negative and positive indicators of mental health in AYAs. A total of 19 studies were included in the meta-analysis, and the results revealed a moderately negative correlation between resilience and negative indicators of mental health (r = - 0.391), and a moderately strong positive correlation with positive indicators of mental health (r = 0.499). Specifically, higher resilience was correlated with reduced levels of perceived stress, anxiety, burnout, and depression, alongside enhanced mental well-being, quality of life, life satisfaction, self-esteem, and self-efficacy among AYAs. These findings are consistent with previous empirical studies covering similar age groups (56, 72–77). Further, in terms of the strength of the correlation, resilience was more strongly correlated with positive indicators of mental health than negative indicators, also supported by the previous evidence (78, 79).

This systematic review provides preliminary evidence on the correlation between resilience and mental health in AYAs. Specifically, this age group encompasses adolescence, from 10 to 19 years old, and emerging adulthood, from 18 to 25 years old, covering a critical developmental stage that spans the transition from education to early social integration. This period is foundational for establishing lifelong health and facilitating personal and professional development (3, 4). Uniquely, this stage is characterized by intensified academic pressure and increased challenges in building and maintaining relationships, which distinguish it from childhood. Meanwhile, the urgency to acquire advanced skills for societal survival exceeds that observed in middle adulthood (80). As a result, this is the period when individuals are most threatened by psychosocial stressors and are vulnerable to mental health disorders (81), which adversely affect academic and occupational achievement, interpersonal relationship formation, self-identity, etc. (36). Over time, untreated mental health disorders during this critical stage may produce long-lasting negative consequences for individuals, including reduced workforce participation, lower incomes, and diminished living standards in later adulthood (82). The more individuals affected, the greater the likelihood of harm at the economic and cultural levels of society.

As highlighted by the World Health Organization, fostering resilience has been an integral part of the strategy for preventing mental health disorders (83), The findings of this study reinforce the significant role of resilience in helping AYAs reduce negative indicators of psychopathology and enhance positive mental health states. These results are supported by relevant empirical studies. In terms of the elimination of negative indicators, resilience has been shown to alleviate symptoms of depression and anxiety in adolescents (84), reduces the after-effects of psychological trauma, and curbs suicidal ideation in young adults (85, 86). Conversely, lower resilience leads to a heightened risk of various mental health disorders (87). In terms of the promotion of positive indicators, resilient adolescent individuals perceive increased life satisfaction (51), possess better emotional regulation to manage negative emotions and prefer positive coping strategies to achieve social adaptation (88). Furthermore, resilience positively affects all dimensions of quality of life (89), while life satisfaction, perceived well-being, and self-efficacy are all negatively affected when resilience is low (90). Although the exact mechanisms that resilience promotes mental health remain complicated (91), resilience theory provides a well-established framework to explain its effects. Resilience facilitates mental health by enabling individuals to counteract the negative consequences of exposure to risk factors. This process involves leveraging environmental, social, and personal protective factors to interrupt the progression from risk exposure to pathological outcomes (33). In specific, through the mobilization of these internal and external protective factors, individuals are able to face up to adverse life events and learn from their struggles to achieve further growth and positive cognitive development (92). As a result, individuals might avoid psychological dysfunction (31), be less likely to suffer from detrimental mental health outcomes (93), and raise the positive indicators such as the perceived well-being (32, 94) to contribute to the promotion of overall mental health (95–97). Additionally, similar positive effects have been explored through theoretical mechanisms. For instance, resilience can promote post-traumatic growth through the mediating effects of internal factors, such as positive emotions, and external factors, such as social support (98). Given the alarming mental health challenges faced by AYAs and the rising demand for effective mental health interventions (99), the results of this study provide a strong referential basis for developing targeted interventions that leverage resilience as a protective and promotive factor for mental health.

This study explored the moderators influencing the correlation between resilience and mental health in AYAs, including percentage of female participants, sample regions, and resilience measurements. First, we found that sample regions moderated the correlation between resilience and positive indicators of mental health, whereas no such moderating effect was observed for negative indicators. Similar results have been found in previous studies, though the specifics vary (100). Our findings revealed that the correlations reported in studies conducted in Western countries were stronger than those from Eastern countries. The perspective of sociocultural differences between Western and Eastern societies may offer a potential explanation for this finding. Current researches and measurements of resilience are predominantly based on cognitive or individual-level characteristics (81), which align more closely with the individualistic cultural context of Western countries (101). In contrast, within the collectivist cultural context of Eastern societies, connections to broader social systems surrounding the individual should also be considered as a key dimension of resilience (102). However, only a limited number of scales in the existing literature have incorporated this aspect. Moreover, this finding may also be attributed to varying levels of awareness of the resilience concept. Greater awareness of resilience tends to foster positive attitudes and adaptive behaviors, which, in turn, strengthen the association between resilience and mental health outcomes (103). Given that the concept of resilience originated in Western contexts and has been studied across disciplines as early as the beginning of the 21st century (20), it is likely more established in Western cultures compared to Eastern ones (101), leading to the present findings of this study. However, in recent years, efforts to localize and adapt the concept of resilience within Eastern cultural frameworks have gained momentum, presenting promising opportunities for advancing cross-cultural resilience research (92, 104, 105).

Second, resilience measurements also moderated the correlations between resilience and positive indicators of mental health in AYAs. Specifically, although the CD-RISC scale was considered the more commonly used instruments for assessing resilience (106), the RS scale yielded higher correlation coefficients in the studies included. One potential explanation is that in terms of applicability to the participant populations, the RS scales (including short versions) are considered to be more appropriate for the adolescent population, as well as possessing cross-age applicability (107). By contrast, the CD-RISC has shown better measurement properties when including general population and clinical samples, making it more applicable in the clinical practice through resilience interventions (16). In addition, the stability of the scales’ factor structure may also serve as a potential explanation for the findings. For instance, the original version of the CD-RISC-25 demonstrated instability in its proposed five-factor structure (16). Many researchers have reported that it is better represented by a three-factor structure (108) or one general factor (109). In contrast, the RS scale has consistently maintained its solid one-factor measure of resilience (81). Concerning the negative indicators of mental health, we did not find a moderating effect of the resilience measurements.

Finally, while prior studies reported that a higher percentage of female participants strengthened the correlation between resilience and mental health (41, 84), this effect was not observed in our analysis when the percentage of female participants was higher (>50%) in either the positive or the negative indicators. This discrepancy may be explained by the limited number of included studies, necessitating cautious interpretation and future research to validate the findings.

## 5 Limitations and future directions

Although conducted in strict accordance with the relevant standards and procedures, the current meta-analysis has several limitations as follows. First, we used only Pearson’s correlation coefficient to measure effect sizes, which limited the number of studies we included in the meta-analysis and thus might affect the validity and generalization of the results. Future studies should consider incorporating other metrics such as phi-coefficient, point-biserial correlation, and Spearman rank-order correlation coefficient as well. Second, potential moderators were not comprehensive enough as we only considered percentage of female participants, sample regions, and resilience measurements. Future studies should take sufficient account of other variables, such as educational attainment, ethnicity, and adversity factors, all of which may influence the relationship between resilience and mental health in AYAs, particularly adversity factors. Finally, our meta-analysis, based mainly on cross-sectional studies, was not able to provide an explanation for the causal relationship between resilience and mental health among AYAs. Therefore, future research should prioritize the use of longitudinal study designs. For instance, tracking resilience and mental health indicators across multiple time points while addressing confounding factors can provide valuable insights into their potential causal relationships.

## 6 Conclusions

The current meta-analysis included 19 studies on the correlation between resilience and mental health in AYAs. Our results indicated that resilience showed a moderately negative correlation with negative indicators of mental health and a moderately strong positive correlation with positive indicators of mental health. Additionally, the strength of the correlation between resilience and positive indicators of mental health was moderated by sample regions and resilience measurements. By extending the application of the two-factor model of mental health, this study demonstrated that previous findings on the correlation between resilience and mental health in other age groups are also applicable to AYAs aged 10-25. This provides a more direct and robust basis for future studies of resilience in this population. Therefore, we advocate for the further exploration of targeted resilience-building interventions and strategies in school and workplace settings, which could effectively help to mitigate the increasing mental health challenges faced by AYAs.
