# Supplementary material for: The correlation between resilience and mental health of adolescents and young adults: a systematic review and meta-analysis
Source: Front Psychiatry. 2025 Feb 10;16:1536553. doi: 10.3389/fpsyt.2025.1536553 (PMC11848722; doi:10.3389/fpsyt.2025.1536553)
Supplement: Supplementary file 1 [file Table1.docx]

Supplementary Material

# Supplementary Tables

# Supplementary Table 1

Search strategy in PubMed

| **Step** | **Search strategy** |
| --- | --- |
| #1 | (("Resilience, psychological"[MeSH Terms] OR ("psychological resilience"[Title/Abstract] OR "Resilience"[Title/Abstract] OR "Resiliences"[Title/Abstract] OR "Resiliency"[Title/Abstract] OR "Resiliencies"[Title/Abstract] OR "resilient response"[Title/Abstract]) OR "stress immunity"[Title/Abstract] OR "recovery"[Title/Abstract] OR "elasticity"[Title/Abstract])) |
| #2 | ("Mental Health"[MeSH Terms] OR "mental hygiene"[Title/Abstract] OR "mental well being"[Title/Abstract] OR "mental health disorders"[Title/Abstract] OR "mental health problems"[Title/Abstract] OR "psychological health"[Title/Abstract] OR "psychological well-being"[Title/Abstract]) |
| #3 | (((((((((((((((((("Adolescent"[Mesh]) OR (Adolescents[Title/Abstract])) OR (Adolescence[Title/Abstract])) OR (Adolescents, Female[Title/Abstract])) OR (Adolescent, Female[Title/Abstract])) OR (Female Adolescent[Title/Abstract])) OR (Female Adolescents[Title/Abstract])) OR (Adolescents, Male[Title/Abstract])) OR (Adolescent, Male[Title/Abstract])) OR (Male Adolescent[Title/Abstract])) OR (Male Adolescents[Title/Abstract])) OR (Youth[Title/Abstract])) OR (Youths[Title/Abstract])) OR (Teens[Title/Abstract])) OR (Teen[Title/Abstract])) OR (Teenagers[Title/Abstract])) OR (Teenager[Title/Abstract])) OR (Young Adults[Title/Abstract])) |
| #4 | ("cross sectional studies"[MeSH Terms] OR ("cross sectional"[All Fields] AND "studies"[All Fields]) OR "cross sectional studies"[All Fields] OR ("cross"[All Fields] AND "sectional"[All Fields] AND "study"[All Fields]) OR "cross sectional study"[All Fields] OR ("cohort studies"[MeSH Terms] OR ("cohort"[All Fields] AND "studies"[All Fields]) OR "cohort studies"[All Fields] OR ("cohort"[All Fields] AND "study"[All Fields]) OR "cohort study"[All Fields]) OR ("longitudinal studies"[MeSH Terms] OR ("longitudinal"[All Fields] AND "studies"[All Fields]) OR "longitudinal studies"[All Fields] OR ("longitudinal"[All Fields] AND "study"[All Fields]) OR "longitudinal study"[All Fields]) OR ("retrospective studies"[MeSH Terms] OR ("retrospective"[All Fields] AND "studies"[All Fields]) OR "retrospective studies"[All Fields] OR ("retrospective"[All Fields] AND "study"[All Fields]) OR "retrospective study"[All Fields]) OR ("prospective studies"[MeSH Terms] OR ("prospective"[All Fields] AND "studies"[All Fields]) OR "prospective studies"[All Fields] OR ("prospective"[All Fields] AND "study"[All Fields]) OR "prospective study"[All Fields]) OR ("case control studies"[MeSH Terms] OR ("case control"[All Fields] AND "studies"[All Fields]) OR "case control studies"[All Fields] OR ("case"[All Fields] AND "control"[All Fields] AND "study"[All Fields]) OR "case control study"[All Fields])) |
| #5 | #1 AND #2 AND #3 AND #4 |

# Supplementary Table 2

The quality assessment of included studies

| Study | Item1 | Item2 | Item3 | Item4 | Item5 | Item6 | Item7 | Item8 | Total |
| --- | --- | --- | --- | --- | --- | --- | --- | --- | --- |
| Achour 2014 | N | N | U | Na | N | N | Y | Y | 25.00% |
| Anyan 2017 | Y | Y | Y | Y | Y | Y | Y | Y | 100.00% |
| Chow 2018 | Y | Y | U | Na | Y | Y | Y | Y | 75.00% |
| de la Fuente 2021 | N | Y | N | Y | N | N | Y | Y | 50.00% |
| Hjemdal 2011 | N | Y | U | Y | Y | Y | Y | Y | 75.00% |
| Ibigbami 2024 | N | Y | Na | Y | Y | Y | Y | Y | 75.00% |
| Hayas 2022 | Y | Y | Y | Y | Y | Y | Y | Y | 100.00% |
| Lau 2022 | Y | Y | Y | Y | N | N | Y | Y | 75.00% |
| Marulanda 2016 | Y | Y | Y | Y | Y | Y | Y | Y | 100.00% |
| Masuyama 2022 | N | N | Y | Y | Y | Y | Y | Y | 75.00% |
| Rayani 2024 | Y | Y | Na | Na | Y | Y | Y | Y | 75.00% |
| Rew 2024 | Y | Y | Y | Y | Y | Y | Y | Y | 100.00% |
| Scheiner 2023 | Y | Y | Y | Y | Y | N | Y | Y | 87.5% |
| Shek 2018 | N | Y | Na | Na | Y | Y | Y | Y | 62.5% |
| Shi 2018 | N | Y | Y | Y | Y | Y | Y | Y | 87.5% |
| Kumar Sia 2024 | N | Na | Na | Na | Y | Y | Y | Y | 50.00% |
| Visier-Alfonso 2024 | Y | Y | Y | Y | Y | Y | Y | Y | 100.00% |
| Zhu 2023 | Y | Y | Y | Y | Y | Y | Y | Y | 100.00% |
| Konaszewski 2021 | U | Y | Y | Y | Y | Y | Y | Y | 87.5% |

Note. Item 1 “Were the criteria for inclusion in the sample clearly defined?”, Item 2 “Were the study subjects and the setting described in detail?”, Item 3 “Was the exposure measured in a valid and reliable way?”, Item 4 “Were objective, standard criteria used for measurement of the condition?”, Item 5 “Were confounding factors identified?”, Item 6 “Were strategies to deal with confounding factors stated?”, Item 7 “Were the outcomes measured in a valid and reliable way?”, Item 8 “Was appropriate statistical analysis used?”; Y, Yes; N, No; U, Unclear; Na, Not applicable.
